# Renewed Response to Pembrolizumab After Salvage Radiotherapy for Oligoprogression in Advanced NSCLC: A Case Report

**DOI:** 10.1002/rcr2.70674

**Published:** 2026-07-14

**Authors:** Takayuki Niitsu, Satoshi Tanaka, Moto Yaga, Makoto Yamamoto, Chika Takahashi, Yoshitaka Usui, Daiki Nagira, Tetsuaki Tsuji, Kento Yokota, Satoshi Tobita, Kiyonobu Ueno

**Affiliations:** ^1^ Department of Respiratory Medicine Osaka General Medical Center Osaka Japan; ^2^ Department of Respiratory Medicine and Clinical Immunology Osaka University Graduate School of Medicine Suita Japan; ^3^ Tanaka Internal Medicine and Respiratory Clinic Osaka Japan

**Keywords:** immune checkpoint inhibitor, non‐small cell lung cancer, oligoprogression, pembrolizumab, rechallenge

## Abstract

Immune checkpoint inhibitor (ICI) rechallenge may benefit selected patients with NSCLC, but the role of local radiotherapy for oligoprogression and late same‐agent rechallenge remains uncertain. We report a patient with Stage IVA lung adenocarcinoma and a programmed death‐ligand 1 tumour proportion score of ≥ 75% who achieved a durable partial response to first‐line pembrolizumab. After isolated left supraclavicular nodal oligoprogression in 2020, he received salvage radiotherapy and remained off systemic treatment for almost 5 years. In late 2025, multiple new right lower lobe nodules and pleural‐based lesions appeared near the original primary site and enlarged on serial CT, prompting pembrolizumab rechallenge. CT in February 2026 showed a renewed partial response. This case suggests that rechallenge with pembrolizumab may remain reasonable at late relapse in carefully selected patients with durable initial benefit, limited progression controlled locally, a long treatment‐free interval, and good performance status.

## Graphical abstract

A patient with PD‐L1‐high advanced NSCLC had a durable benefit from first‐line pembrolizumab, underwent salvage radiotherapy for isolated nodal oligoprogression, and remained off systemic treatment for almost 5 years. At late relapse, the same agent, pembrolizumab, was rechallenged and again produced a partial response, suggesting that rechallenge may be reasonable in carefully selected patients.

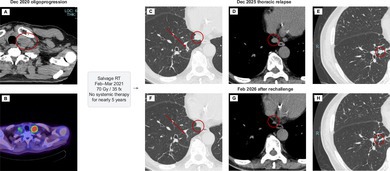

## 1 Introduction

Immune checkpoint inhibitors (ICIs) are a standard treatment for advanced NSCLC without sensitising EGFR mutations or ALK rearrangements and are also used for unresectable locally advanced disease and perioperative therapy.

Recent studies have reported that ICI rechallenge may be reasonable in selected patients [1]. However, available reports are heterogeneous, and the benefit of ICI rechallenge remains difficult to predict.

Another practical challenge is limited progression during otherwise effective ICI therapy. Retrospective studies suggest that local radiotherapy can prolong disease control in selected patients with oligoprogression, but the evidence is heterogeneous [2].

We report a patient with advanced NSCLC who had a long‐lasting response to first‐line pembrolizumab, received radiotherapy for isolated left supraclavicular nodal oligoprogression, remained off systemic treatment for almost 5 years, and then again achieved a partial response to pembrolizumab rechallenge.

## 2 Case Report

A 68‐year‐old former smoker with chronic obstructive pulmonary disease, diabetes mellitus, and an Eastern Cooperative Oncology Group performance status (ECOG PS) of 1 presented with haemoptysis in July 2017. Computed tomography (CT) showed a 7.0 × 5.5 cm cavitary irregular mass in the right lower lobe, enlarged right hilar, mediastinal, and right supraclavicular lymph nodes, and a low‐attenuation lesion in hepatic Segment 7 (Figure 1A–D). PET‐CT showed uptake in the lung, nodal, and liver lesions. Brain MRI showed no metastases. Transbronchial biopsy revealed adenocarcinoma. Tumour cells were positive for thyroid transcription factor‐1 and Napsin A and negative for CK14 and p40. EGFR mutation and ALK rearrangement testing were negative. The programmed death‐ligand 1 tumour proportion score was ≥ 75%. He was diagnosed with right lower lobe adenocarcinoma (cT3N3M1b, Stage IVA) with nodal and liver metastases.

**FIGURE 1:**
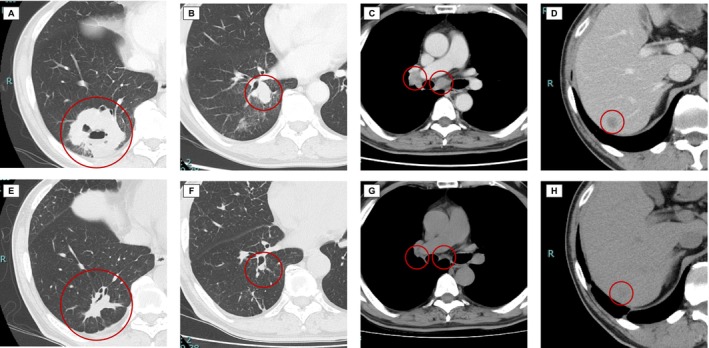
Initial disease and response to first‐course pembrolizumab. Chest and abdominal CT at presentation in July 2017 showing a 7.0 × 5.5 cm cavitary irregular mass in the right lower lobe (A), right hilar/peribronchial disease (B), mediastinal/hilar nodal disease (C), and a hepatic Segment 7 lesion (D). Chest and abdominal CT in November 2017 showing shrinkage of each target lesion (E–H).

Pembrolizumab monotherapy was started in August 2017. The primary lesion, nodal disease, and liver metastasis regressed, with a partial response maintained on serial imaging (Figure 1E–H). He received 52 cycles through November 2020 at his request. He developed immune‐related thyroid dysfunction, with transient hyperthyroidism followed by hypothyroidism, controlled with levothyroxine.

Surveillance CT in September 2020 showed enlargement of a left supraclavicular lymph node, whereas the primary lesion remained shrunken and no new distant lesions were detected. Repeat CT in December 2020 confirmed further nodal growth (Figure 2A). PET‐CT (Figure 2B), fine‐needle aspiration, and nodal biopsy confirmed adenocarcinoma. Because progression was limited to a single biopsy‐proven site, with continued control of the primary tumour and all other metastatic sites under otherwise effective pembrolizumab therapy, this pattern was considered oligoprogression, consistent with definitions describing progression at one or a few sites in the setting of otherwise controlled systemic disease. Salvage radiotherapy to the left supraclavicular node was delivered from February to March 2021 (70 Gy in 35 fractions). The treated node regressed, and follow‐up imaging showed continued control of the thoracic and hepatic disease. No systemic treatment was given after radiotherapy.

**FIGURE 2:**
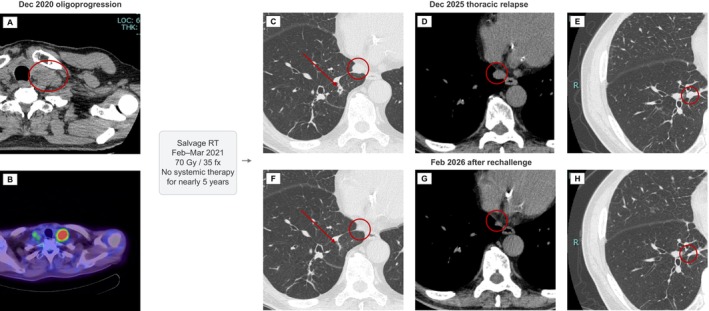
Nodal oligoprogression, salvage radiotherapy, and late pembrolizumab rechallenge. Chest CT in December 2020 showing enlargement of the left supraclavicular lymph node while the primary lesion remained controlled (A) and PET‐CT showing uptake in the left supraclavicular lesion (B). Chest CT in December 2025 showing new and enlarging right lower lobe nodules and pleural‐based lesions near the original primary site (C–E), and chest CT in February 2026 showing partial response after pembrolizumab rechallenge (F–H).

CT in September 2025 showed multiple new right lower lobe nodules and pleural‐based lesions near the original primary site, and repeat CT 3 months later showed further enlargement (Figure 2C–E). PET‐CT was not performed, and these lesions were not biopsied. Therefore, the event was regarded as a thoracic relapse on clinical and radiologic grounds.

His ECOG PS was 0. Given the prior durable benefit from pembrolizumab, the nearly 5‐year treatment‐free interval after local therapy, and multifocal thoracic progression not readily addressable with local treatment alone, pembrolizumab rechallenge was initiated in December 2025. CT in February 2026 showed a partial response (Figure 2F–H).

## 3 Discussion

This case highlights two clinically important treatment decisions. First, local radiotherapy for oligoprogression after a durable pembrolizumab response. Second, late same‐agent rechallenge after a long treatment‐free interval.

The first progression was considered oligoprogression because it was limited to a single biopsy‐proven left supraclavicular lymph node while the primary lesion and other metastatic sites remained controlled. Salvage radiotherapy was therefore selected, although evidence for this approach remains largely retrospective [2]. In contrast, the 2025 event consisted of multiple new and enlarging thoracic lesions after almost 5 years off systemic treatment. Because the pattern was no longer a single‐site event amenable to local treatment alone, and because the patient had maintained good performance status and had previously derived prolonged benefit from pembrolizumab, rechallenge was considered reasonable.

Evidence for ICI rechallenge depends on the clinical context. Prospective data mainly support second‐course pembrolizumab after protocol‐defined treatment completion, whereas outcomes are less consistent after progression during active ICI therapy [3]. Rechallenge after local therapy has also been described, but usually early after local treatment [1, 4].

Our patient differed from these cohorts in that pembrolizumab rechallenge was not initiated immediately after salvage radiotherapy, and a renewed partial response was observed after an almost 5‐year treatment‐free interval. Although the mechanism remains unproven, prior radiotherapy may have enhanced antigen release and presentation, interferon‐related signalling, and systemic T‐cell priming, thereby making late renewed sensitivity to PD‐1 blockade biologically plausible [5].

This report has important limitations. PET‐CT and biopsy were not performed for the late thoracic lesions; therefore, a second primary lung cancer or benign inflammatory lesions cannot be excluded. However, serial CT showed multiple new lesions near the original primary site that continued to enlarge over 3 months, making malignant relapse more likely. An ICI‐related sarcoid‐like reaction was considered less likely because it usually occurs during active treatment and commonly involves mediastinal or hilar lymph nodes.

In conclusion, pembrolizumab rechallenge may still be reasonable in carefully selected patients with durable initial benefit, limited progression controlled with local radiotherapy, a long treatment‐free interval, and good performance status.

## Author Contributions

Takayuki Niitsu drafted the manuscript. Satoshi Tanaka, Moto Yaga, and Makoto Yamamoto critically revised the manuscript. Takayuki Niitsu and Satoshi Tanaka managed the patient. All authors have reviewed the manuscript for intellectual content. All authors approved the final version of the manuscript.

## Funding

This work was supported by the Japan Science and Technology Agency (JST) (Grant Number: JPMJAX242F).

## Consent

The authors declare that written informed consent was obtained for the publication of this manuscript and accompanying images using the consent form provided by the Journal.

## Conflicts of Interest

The authors declare no conflicts of interest.

## Data Availability

Data sharing is not applicable to this article as no datasets were generated or analysed during the current study.
